# Celiac disease associated with aplastic anemia in a 6-year-old girl: a case report and review of the literature

**DOI:** 10.1186/s13256-017-1527-5

**Published:** 2018-01-23

**Authors:** Omar Irfan, Sana Mahmood, Heera Nand, Gaffar Billoo

**Affiliations:** 1 Medical College, Aga Khan University Hospital, Karachi, Pakistan; 2 Department of Pediatrics, Aga Khan University Hospital, Karachi, Pakistan

**Keywords:** Aplastic anemia, Celiac disease, Gluten-free diet, Child, Bone transplant

## Abstract

**Background:**

Celiac disease may present with hematological abnormalities including long-standing anemia. Both aplastic anemia and celiac disease have a similar underlying autoimmune process but an association between the two is seldom reported. There have only been three pediatric cases reporting this association and this case is the first reported in a female pediatric patient.

**Case presentation:**

We report a case of 6-year-old South Asian girl presenting with bruises, petechiae, and recent history of loose stools. On evaluation, she was diagnosed as having celiac disease and was put on a gluten-free diet and further investigations including bone marrow biopsy revealed pancytopenia. She was managed with packed red cells, platelets, and diet restrictions and had improving platelet counts over yearly follow ups. Her parents were counseled regarding the need for bone marrow transplant.

**Conclusions:**

This is the fourth case report suggesting an association between celiac disease and aplastic anemia in the pediatric population and this association could be more common than expected. Timely intervention of either celiac disease through strict gluten-free diet or aplastic anemia through immunosuppressive therapy could potentially reduce the risk for other autoimmune conditions. We can see that all four pediatric cases reported with this potential association are from South East Asia and hence larger studies would be prudent to explore this association.

## Background

Celiac disease (CD) is an autoimmune disease characterized by reversible small bowel mucosal inflammation with villous atrophy causing malabsorption [1]. It causes malabsorption of iron, folic acid, and/or vitamin B12, with additional features of anemia of chronic disease [2]. Many hematological manifestations, mostly due to nutritional deficiencies, have been associated with CD [2]; however, the occurrence of aplastic anemia (AA) is extremely rare. There is a known association between CD and AA as both have a similar pathological process involving autoreactive T cell destruction of tissue [3]. Both diseases share an underlying immune mechanism as the human leukocyte antigen (HLA) DQ2 allele is identified in 90 to 95% of celiac individuals, and HLA DQ8 in the rest [1]. The cumulative risk for development of various autoimmune diseases is 8.1% at 15 years of age in patients with CD [4]. Previously, there have been three reported pediatric cases of AA in association with CD as compared to more data for adults [5]. We report the case of a 6-year-old girl with CD and AA causing bone marrow failure. This is the fourth report in the literature to date that suggests an association of AA with CD in the pediatric population.

## Case presentation

A 6-year-old South Asian girl presented in October 2013 with generalized bruises, undocumented fever, and eczematous rashes of 1 month’s duration. She had had six or seven episodes of loose stools per day for 3 months accompanied by loss of appetite. She was a known asthmatic. Her family history was unknown as she was an adopted child. Her foster parents did not notice any food allergens. On examination, she was short with a height of 98 cm (<5th percentile) and weight of 13.5 kg (< 5th percentile). There was pallor, multiple generalized petechiae, and eczematous patches on upper back and behind her ears with typical Fanconi facies including microcephaly and microphthalmia. A systemic examination was normal and no lymphadenopathy was appreciated.

Investigations revealed hemoglobin of 9.0 g/dl, platelet count of 67 × 10^9^/L, and mean corpuscular volume (MCV) and mean corpuscular hemoglobin (MCH) of 75.3 fl and 24.2 pg, respectively with no atypical cells. A liver function test (LFT), renal function test (RFT), urine examination, and coagulation profile were normal and blood culture was sterile. Ultrasonography of her abdomen and portal venous Doppler were normal. Antinuclear antibodies (ANA) titer was negative. Mitomycin C was used to detect chromosomal breakages to rule out Fanconi anemia and results were negative with 0.58 breaks/cell. Investigations for malabsorption revealed gamma A immunoglobulin (IgA) tissue transglutaminase levels to be 370 IU/ml (normal < 12 IU/ml), anti-gliadin antibodies to be 140 IU/ml (normal < 12 IU/ml), but normal thyroid profile. A jejunal biopsy showed complete villous atrophy with increased intraepithelial lymphocytes consistent with diagnosis of CD. She was managed with antibiotics, packed red cells, and platelet support and was put on a gluten-free diet. She was supplemented with multivitamins, vitamin C, and iron. She was discharged on day 17 of hospital stay with hemoglobin of 7.9 g/l, total leukocyte count of 2.5 × 10^9^/l, and platelet count of 46 × 10^9^/l. A bone marrow biopsy was advised. The household was noncompliant to follow ups with yearly follow ups showing varying platelet levels from 34 × 10^9^/L to 124 × 10^9^/L.

The child presented in October 2016 with nonproductive cough, fever, respiratory distress for 2 months, and a history of recurrent infections over the past year. Her hemoglobin was 7.5 g/l, total leukocyte count of 3.5 × 10^9^/l, and platelet count of 80 × 10^9^/l. High-resolution computed tomography (HRCT) was suggestive of a lung abscess. Lung culture revealed *Pseudomonas aeruginosa*. GeneXpert, galactomannan, and β-d-glucan (BDG) antibodies were negative. She was managed symptomatically with blood transfusions and platelet support. A bone marrow trephine biopsy was done after consent that revealed hypocellular bone marrow with decreased lymphoid cells, plasma cells, erythroid and myeloid precursors with occasional megakaryocytes. We advised that the child have a bone marrow transplantation but the family refused due to financial constraints. The parents were counseled about the nature of the disease and treatment modalities. She is managed with packed red cells, platelet support, and a gluten-free diet; she showed an increasing trend in platelet count on last follow up.

Table 1 shows a timeline for our patient’s past medical history and follow-up visits as well as interventions.

**Table 1:** Timeline of patient’s medical history

	Past medical history		
	A 6-year-old girl with history of bruising and fever for 1 month and loose stools for 3 months. She was a known asthmatic. Her family history was unknown as she was an adopted child.		
	Hospital/out-patient visits	Diagnostic tests	Interventions
October 2013	Presented with bruises, undocumented fever, and eczematous rashes for 1 month. Petechiae all over the body. Loose stools daily for 3 months with loss of appetite.	Hb 9.0 g/dl, platelet 67 × 10^9^/L, MCV 75.3 fl, MCH 24.2 pg. Normal LFT, RFT, urine examination, and coagulation profile. Blood culture was sterile. ANA titer was negative. Chromosomal breakages was negative with 0.58 breaks/cell, IgA tTG 370 IU/ml, anti-gliadin antibodies 140 IU/ml. Jejunal biopsy – complete villous atrophy with increased intraepithelial lymphocytes.	Antibiotics, packed red cells, and platelet support given. Put on a gluten-free diet, supplemented with multivitamins, vitamin C, and iron. She was discharged on day 17 of hospital stay with Hb 7.9 g/l, TLC 2.5 × 10^9^/l, platelet count of 46 × 10^9^/l, and gluten-free diet. Bone marrow biopsy was advised.
	Noncompliant with yearly follow ups.	Varying platelet levels from 34 × 10^9^/L to 124 × 10^9^/L.	Gluten-free diet
October 2016	Nonproductive cough, fever, respiratory distress for 2 months. History of recurrent infections over the year	Lung abscess and a cavitary lesion seen on HRCT. Lung culture revealed *Pseudomonas aeruginosa*. GeneXpert, galactomannan, and BDG antibodies were negative. A bone marrow trephine biopsy revealed hypocellular bone marrow.	She is planned for bone marrow transplantation. Parents were counseled about the disease and treatment. Managed with packed red cells, platelet support, and a gluten-free diet. She showed an increasing trend in platelet count.

*ANA* antinuclear antibodies, *BDG* β-d-glucan, *Hb* hemoglobin, *HRCT* high-resolution computed tomography, *LFT* liver function test *MCH* mean corpuscular hemoglobin, *MCV* mean corpuscular volume, *RFT* renal function test, *TLC* total leukocyte count, *tTG* tissue transglutaminase

## Discussion

CD usually presents with long-standing anemia that may [3] be the only presenting symptom. Other hematological abnormalities include thrombocytosis, leukopenia, thromboembolism, and increased bleeding tendency. There may also be IgA deficiency, hyposplenism, and lymphoma. However, an association between CD and AA is seldom reported [6]. CD should always be considered in patients with hematological abnormalities even without any gastrointestinal manifestations (atypical CD). Diarrhea as a reported symptom is less frequent than before [7].

Table 2 compares the studies done on the CD–AA association.

**Table 2:** Comparison of all studies on patients with aplastic anemia and celiac disease

Study	Grey-Davies and colleagues [3]	Salmeron and colleagues [12]	Maheshwari and colleagues [8]	Kumar and colleagues [9]	Badyal and colleagues [5]	Present study 2017
No. of cases	3	5	1	1	1	1
Age (years)	47, 37, 23	NA	13	10	9	6
Gender	All female	NA	Male	Male	Male	Female
Country	England	England	India	India	India	Pakistan
Infection	One patient had *Aspergillus* and *Pseudomonas* infection and died	NA	No history of infection present	No history of infection present	No history of infection present	Had infection with *Pseudomonas* recently with a history of infections.
Small intestinal biopsy	Partial, subtotal, and total villous atrophy	Subtotal villous atrophy	Total villous atrophy	Subtotal villous atrophy	Biopsy not done because of active GI bleed and low platelet counts	Total villous atrophy with increased intraepithelial lymphocytes
IgA-tTG levels	Not mentioned	NA	>205 IU/mL	100 IU/mL	>105 IU/mL	370 IU/mL
Bone marrow examination and biopsy	Aplastic anemia	Aplastic anemia	Aplastic anemia	Not done	Aplastic anemia	Aplastic anemia

*NA*, not available; *IgA*, gamma A immunoglobulin; *IgA -tTG*, gamma A immunoglobulin tissue transglutaminase; *GI*, gastrointestinal; *IU/ml*, International units per millilitre

A total of 11 cases with CD–AA association have been reported in the literature, out of which three are pediatric cases. In two of these three pediatric cases, both diseases were diagnosed simultaneously but in the third patient the diagnosis of AA is unconfirmed as no bone marrow biopsy was performed [8, 9]. The most recent case developed AA after a previous diagnosis of CD [5]. There have been eight adult cases reported out of which five were diagnosed with CD and AA simultaneously and three had CD previously [5]. The adult ages ranged from 23 to 47 years while the pediatric cases ranged from ages 9 to 13 years. All the adults were female while all the pediatric cases were male. We report the fourth case in the pediatric population and the first female pediatric patient with a CD–AA association. All four pediatric cases reported, including ours, are from India or Pakistan.

The mechanism of cause and effect regarding this is unclear. CD is an autoimmune condition that involves gluten exposure and it affects multiple organs like the bone marrow. Micronutrient deficiencies like iron, folic acid, and others, which occur due to malabsorption, may lead to the bone marrow hypoplasia. Hence, a gluten-free diet, micronutrient supplementation, and immunosuppressive techniques like glucocorticoids have a possible beneficial effect on bone marrow function. This is useful for patients from a lower socioeconomic class who find it difficult to afford bone marrow transplants or antithymocyte globulin therapy [10].

All adult patients in a case series by Grey-Davies *et al*. were symptom-free from a gastrointestinal standpoint while adhering to a gluten-free diet [3]. A gluten-free diet decreases the exposure to antigenic stimuli that lead to bone marrow suppression and reverts the process [8]. Our patient was put on a gluten-free diet but due to infrequent follow ups it is difficult to comment on her dietary adherence. Timely immunosuppressive therapy for AA would also regulate development of other autoimmune diseases like CD [3]. There is a report of stabilization of CD in a pediatric patient after allogeneic hematopoietic stem cell transplantation [11] which further strengthens our decision to advise transplantation. It is likely that bone marrow transplant would improve her prognosis.

## Conclusions

This is the fourth case report suggesting an association between CD and AA in children making it probable that an association between these two entities might be more common than expected. It is still debatable whether there should be a call for routine immunological testing for tissue transglutaminase antibodies in patients with AA. There is a need for studies focusing on this association since it has been a nonentity until recently [8]. All four pediatric cases regarding the CD–AA association have been reported from India or Pakistan as evident from Table 2. However, it is not possible to make a final statement linking the two on the data we have to date. This is why we believe there need to be larger studies to further explore this potential association.

## Abbreviations

AA: Aplastic anemia
CD: Celiac disease
